# Data on the vegetative growth at post acclimatization stage of two *dendrobium* genotypes as an effect of different growing media

**DOI:** 10.1016/j.dib.2019.104493

**Published:** 2019-09-11

**Authors:** Yayat Rochayat Suradinata, Murgayanti, Erni Suminar, Anne Nuraini, Jajang Sauman Hamdani, Syariful Mubarok

**Affiliations:** Department of Agronomy, Faculty of Agriculture, Universitas Padjadjaran, Bandung, 45363, Indonesia

## Abstract

The growing medium is an important factor for plant growth and development. Many growing media are used for orchids, but their availability is limited and some are prohibitively expensive. Therefore, alternative growing media need to be studied. This study was conducted to investigate the potency of some alternative growing media for growing two Dendrobium genotypes, *D. sylvanum* and *D. nindii* x *D. stratiotes*, at the post-acclimatization stage. Five growing media were used in this experiment, namely tree fern fibers, coconut fibers, sphagnum moss, asplenium root, and calliandra humus.

**Table:** Specifications Table

Subject area	*Agriculture*
More specific subject area	*Horticulture*
Type of data	*Figures and table*
How data was acquired	*Advanced Bench* pH *Meter 3510,* HTC-2 digital thermo-hygrometer
Data format	*Raw and Analyzed*
Experimental factors	Six months old of *D. nindii* x *D. Stratiotes* and *D. sylvanum* were used. Those orchids have the criteria of 4.8 cm in plant height, 7–8 of leaves number, and it has one shoot number. Five different growing media were used namely coconut chips, asplenium root, calliandra humus, tree fern and sphagnum moss.
Experimental features	*Determination of data of micro-climate, physical properties of growing media and vegetative plant growth*
Data source location	*Sumedang, Indonesia.*
Data accessibility	*The data are obtainable within this article and publicly accessible.*

**Table:** 

**Value of the data** • The data obtained here will contribute to our understanding of use of alternative growing media for orchid • The data could be used for practitioner and as basic data of further research. • The data has an additional value in choosing alterative growing media for orchid

## 1 Data

**Fig. 1:**
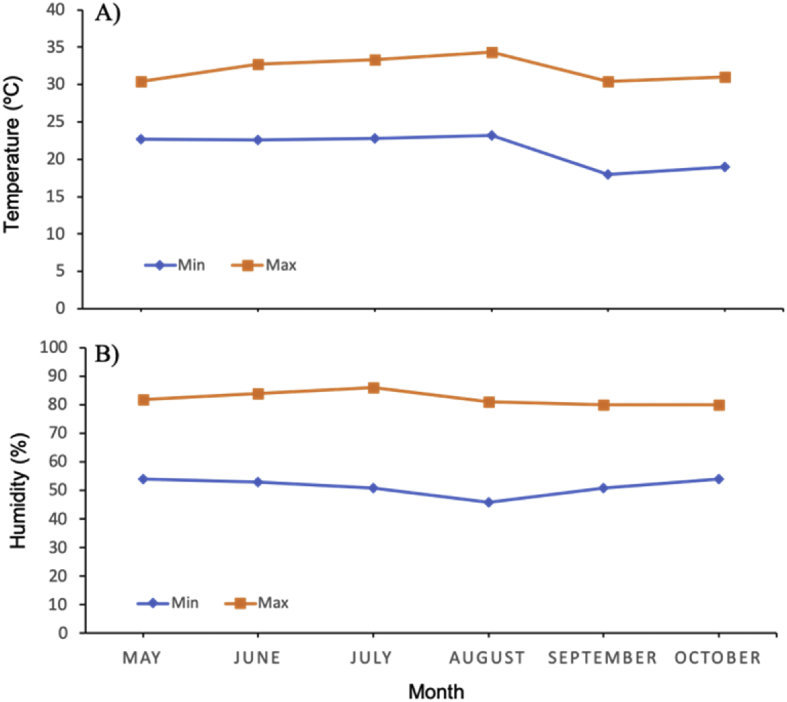
Micro-climate data A) Temperature and B) Humidity during six months of experimental period. Data indicated the average value of minimum and maximum temperature and humidity of each month.

**Fig. 2:**
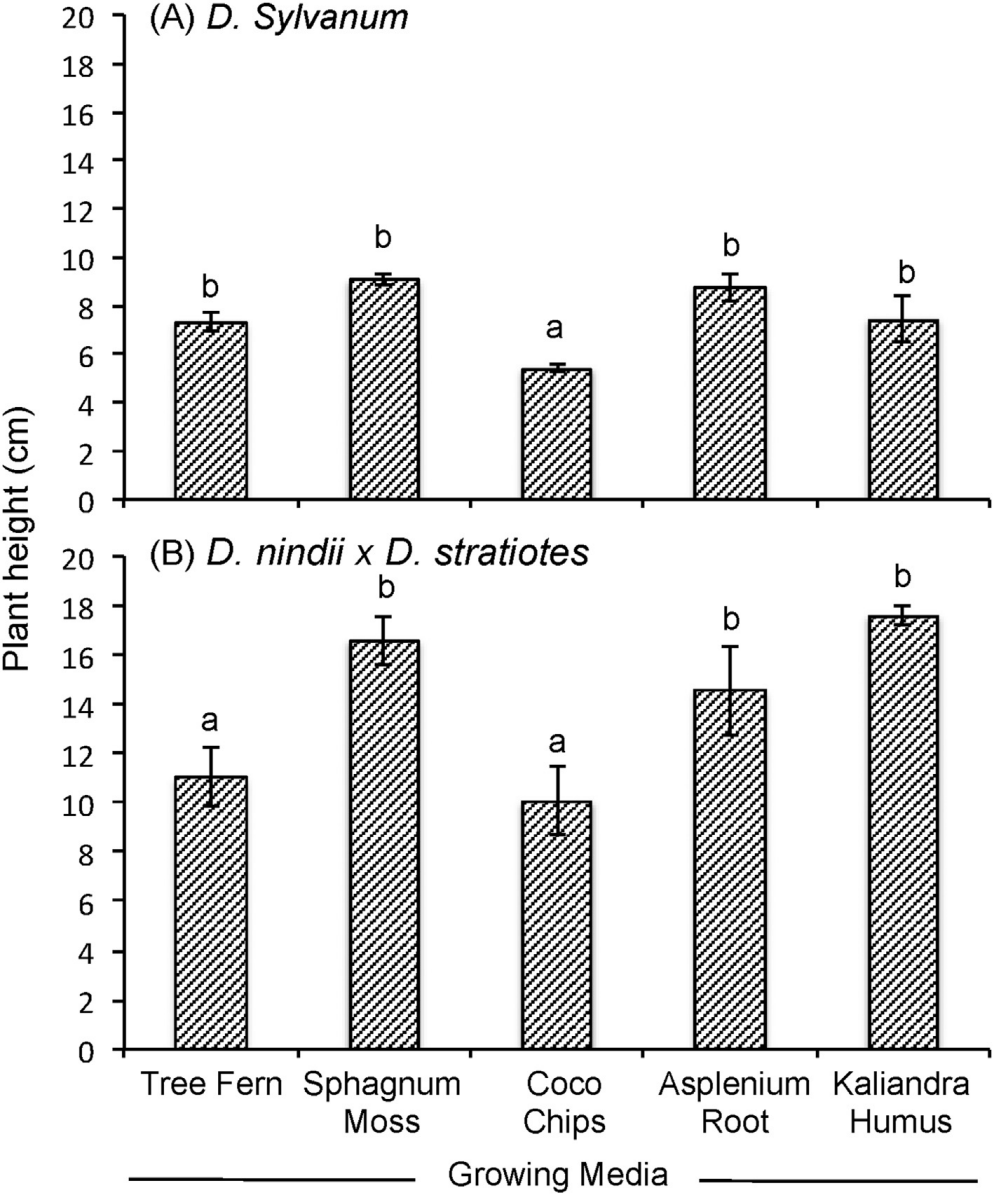
The effect of growing media on the plant height of two dendrobium genotypes. The mean values ± SE (3 replicates) followed by the same lowercase are not significantly different based on Duncan's Multiple Range Test at p < 0.05.

**Fig. 3:**
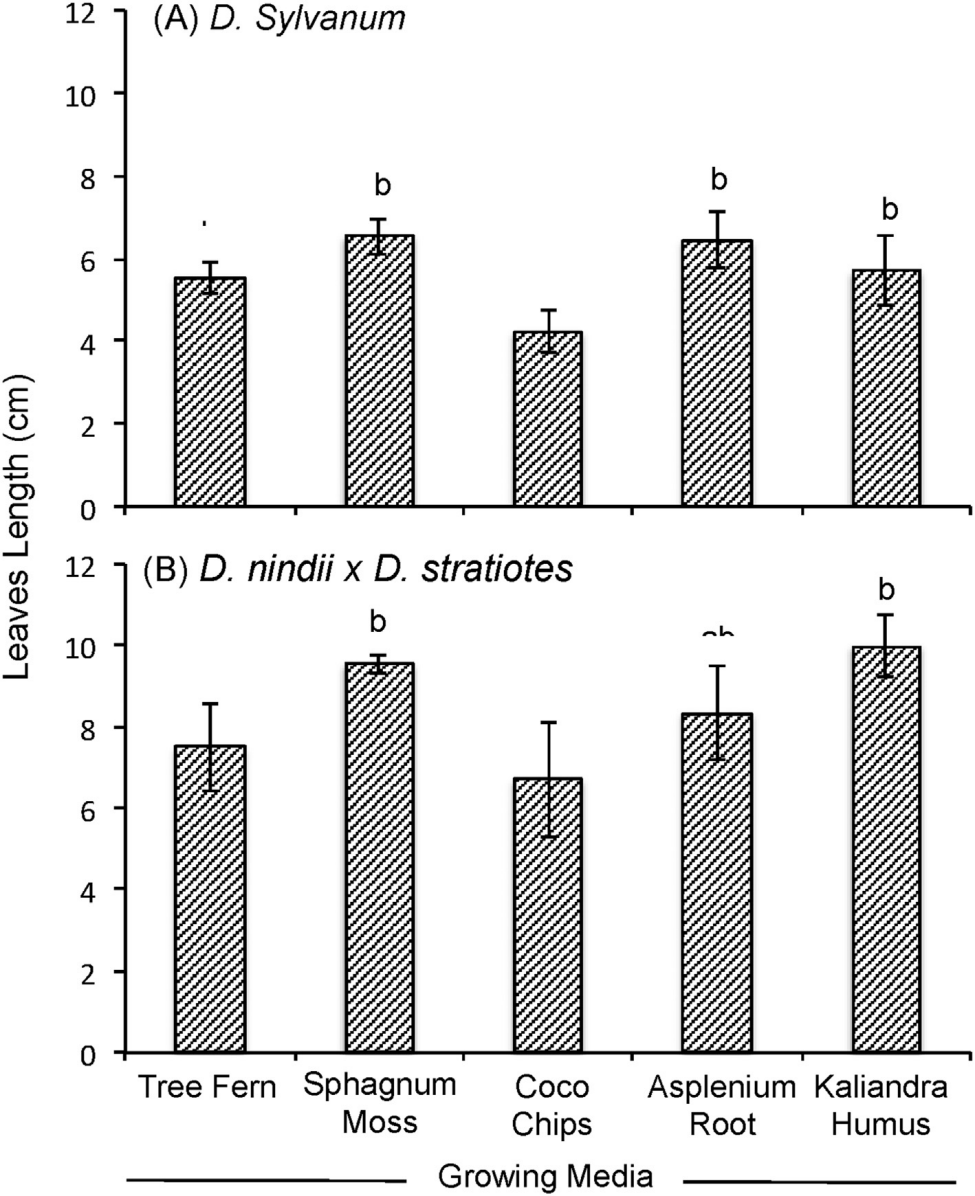
The effect of growing media on leaves length of two dendrobium genotypes. The mean values ± SE (3 replicates) followed by the same lowercase are not significantly different based on Duncan's Multiple Range Test at p < 0.05.

**Fig. 4:**
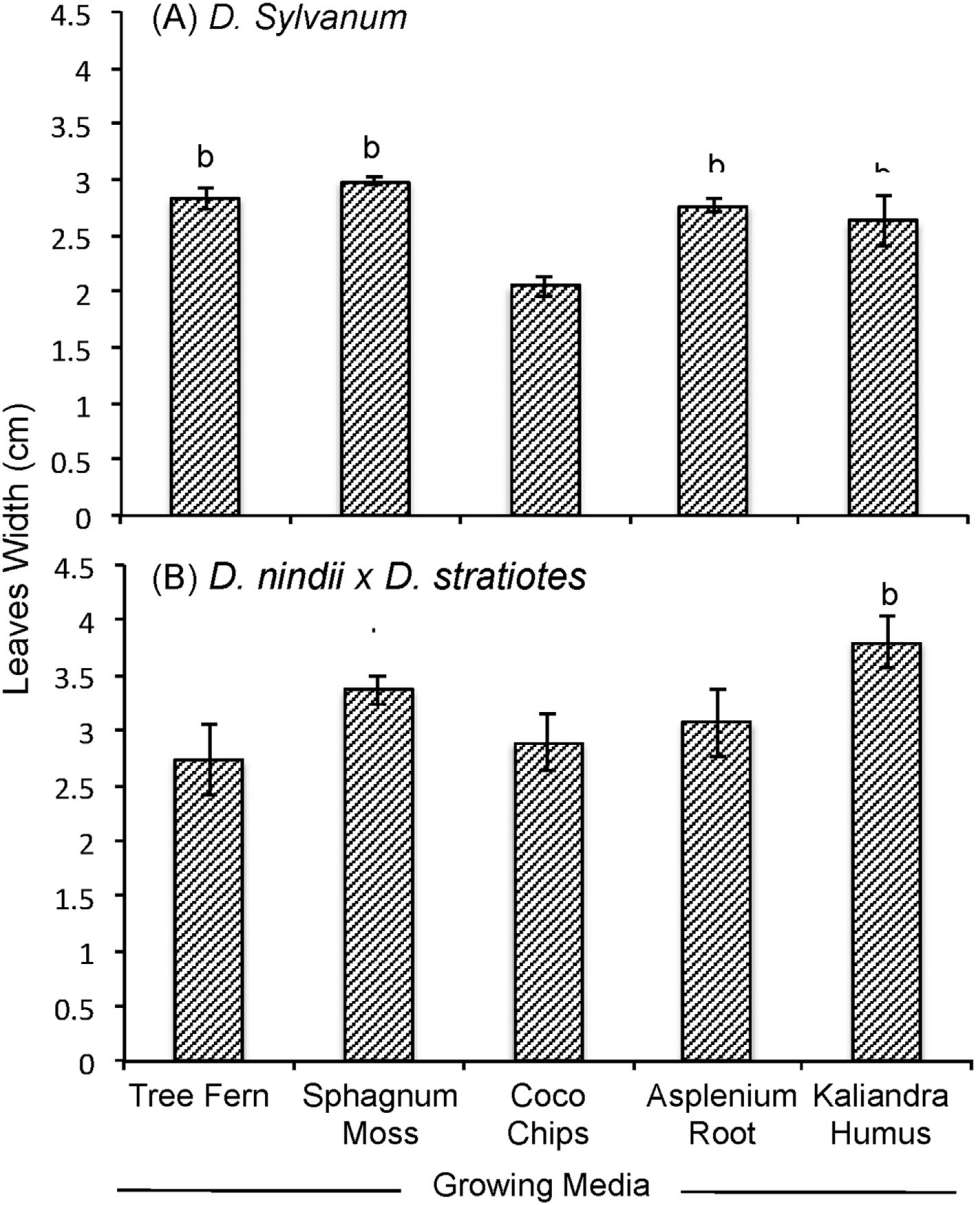
The effect of growing media on leaves width of two dendrobium genotypes. The mean values ± SE (3 replicates) followed by the same lowercase are not significantly different based on Duncan's Multiple Range Test at p < 0.05.

**Fig. 5:**
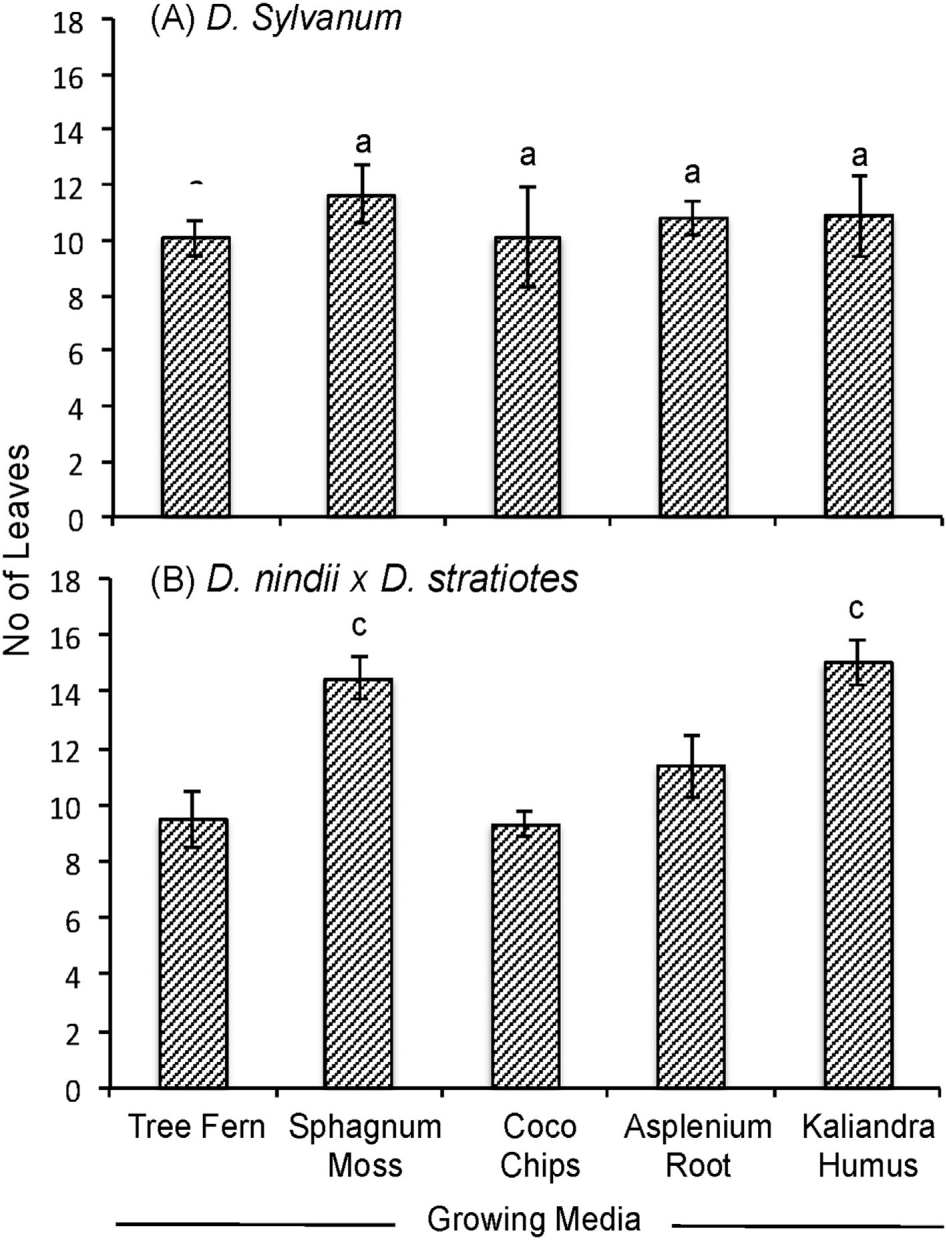
The effect of growing media on no of leaves of two dendrobium genotypes. The mean values ± SE (3 replicates) followed by the same lowercase are not significantly different based on Duncan's at p < 0.05.

**Fig. 6:**
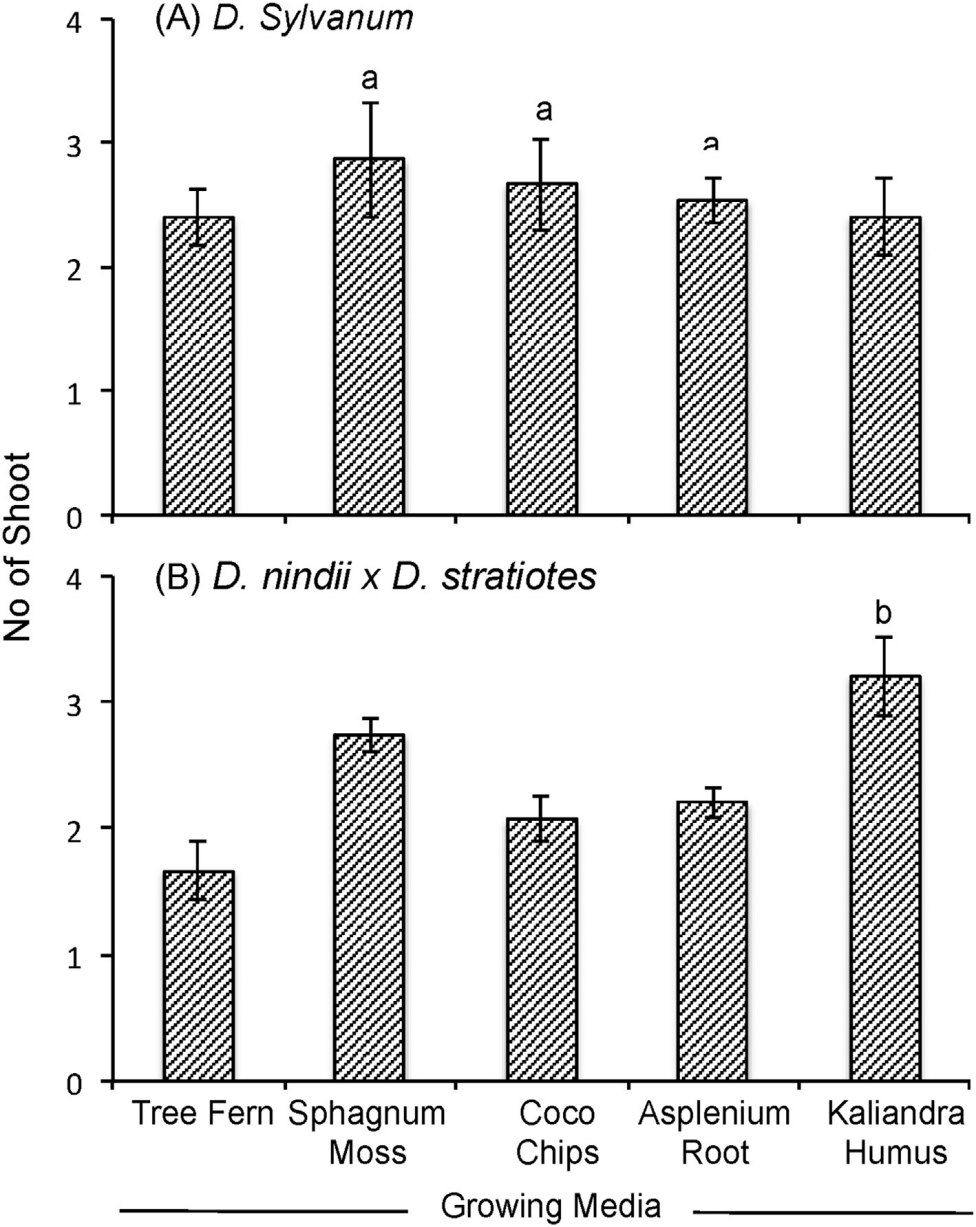
The effect of growing media on the number of shoot of two dendrobium genotypes. The mean values ± SE (3 replicates) followed by the same lowercase are not significantly different based on Duncan's at p < 0.05.

The data report the plant growth analysis of two dendrobium species as an effect of different of growing media. Several parameters related to micro-climate, plant growth (plant height, leaves length, leaves width, number of leaves, number of shoot) and physical properties of growing media (density, porosity, aeration porosity, water holding capacity, stability and pH) were measured. Data on micro-climate data is presented in Fig. 1. Data on plant height, leaves length, leaves width, number of leaves, number of shoots are presented in Fig. 2, Fig. 3, Fig. 4, Fig. 5 and Fig. 6, respectively. Table 1 shows physical properties of five different growing media.

**Table 1:** Physical properties of five different growing media.

Growing Medium	Density (Kg/L)	Porosity (%)	Aeration Porosity (%)	Water Holding Capacity (%)	Stability (%)	pH
Tree Fern	0.153	55	40	15	100	4.58
Sphagnum Moss	0.243	75	35	40	95	5.70
Coconut chips	0.128	30	15	15	100	4.86
Asplenium root	0.075	60	25	35	70	4.52
Calliandra Humus	0.128	50	35	15	100	6.14

## 2 Experimental design, materials, and methods

### 2.1 Plant material and media preparation

Six months old of *D. nindii* x *D. Stratiotes* and *D. sylvanum* were used. Those orchids have the criteria of 4.8 cm in plant height, 7–8 of leaves number, and it has one shoot number. Five different growing media were used namely coconut chips, asplenium root, calliandra humus, tree fern and aphagnum moss that have been obtained from orchid Farmer in Lembang, Bandung, Indonesia. Tree fern, coconut chips and asplenium root were sterilized by boiled in 100 °C for 5 min and then immersed in (Al_2_(SO_4_)3.24H_2_O) for 30 minutes to decrease tannin content as a toxicant. Sphagnum moss was immersed in water for 30 minutes and Calliandra humus was dried before usage. Orchids were removed and transferred from 5 cm pot in diameter to a new 10 cm pot in diameter with a new selected growing media. After that they were placed in green house of Faculty of Agriculture, Universitas Padjadjaran, Indonesia from May 2018 to October 2018. HTC-2 digital thermo-hygrometer (HTC Instruments, India) was used to monitor temperature and humidity during experimental period according to Mubarok et al. [1]. Watering, fertilizing, pest and disease control were done during the experiment periods.

### 2.2 Measurement of physical properties of growing media

Density of growing media was calculated using the standard procedure described by Blake et al. [2] and Chapman [3]. Total porosity was measured by modifying the method described by Boyle et al. [4], where the total porosity was calculated as the ratio of the volume of saturated medium water to the volume of growing media in percentage. Water holding capacity was calculated as the ratio of volume of the drained medium water volume to the volume of growing media in percentage, whereas aeration porosity was calculated as the difference between total porosity and water holding capacity [4].

### 2.3 Plant growth analysis

Plant growth assessments were measured at sixty weeks after replanting (WAP) before re-planted to the bigger pots. Plant height (cm) was measured from the stem base to the tip of the highest leaves, and Leaf length (cm) was measured from the leaf base to the tip of the highest leaves. Leaves wide (cm) was measured on the widest leaf. Leaf number was counted from the accumulation of number of fully opened leaf. Shoot number was determined from the accumulation number of shoots in each plant.

### 2.4 Statistical data analysis

Completely randomized design with four replicates was used for this experiment. For statistical data analysis, data were tested for the normality followed by one factor analysis of variance (ANOVA) was conducted to analyze the data followed by the Duncan's multiple range test at p < 0.05 to compare differences among the growing media.

## References

[1] S. Mubarok, F.F. Farhah, Anas, N. Suwali, D. Kurnia, Kusumiyati, E. Suminar, H. Ezura, Data on the yield and quality of organicallyhybrids of tropical tomato fruits at two stages offruit maturation. Data in Brief. 25 (104031). 10.1016/j.dib.2019.104031 PMC6586725 31249850

[2] Blake G.R., Black C.A., Evans D.D., Ensminger L.E., White J.L., Clark F.E. (1965). Bulk density. Methods of Soil Analysis. Part 1. Physical and Mineralogical Properties, Including Statistics of Measurement and Sampling.

[3] Chapman H.D., Black C.A., Evans D.D., Ensminger L.E., White J.L., Clark F.E. (1965). Cation-exchange capacity. Methods of Soil Analysis. Part 2. Chemical and Microbiological Properties.

[4] Boyle T.H., Craker L.E., Simon J.E. (1991). Growing medium and fertilization regime influence growth and essential oil content of rosemary. Hortscience.

